# Railway Surgery

**Published:** 1899-05

**Authors:** 


### Book Reviews.

Railway Surgery. A Handbook on the Management of Injuries. By Clinton B. Herrick, M. D., Troy, N. Y., Lecturer in Clinical Surgery, Albany Medical College; Attending Surgeon to the Troy Hospital and the House of Good Shepherd; Consulting Surgeon to the Leonard Hospital; Surgeon to the Delaware and Hudson and the Fitchburg Railways; President of the New York State Association of Railway Surgeons. New York: William Wood & Co. 1899.

The Treatment of Wounds. Its principles and practice, general and special. By Lewis Stephen Pilcher, A. M., M. D., Surgeon to Methodist Episcopal Hospital, New York; late passed Assistant Surgeon, U. S. N., Fellow of the American Surgical Society, etc., etc. With 142 engravings New York: William Wood & Co. 1899.

It is seldom that a publishing house puts forth two volumes which are so unlike, yet so companionable. Here are two works on surgery which are well-nigh indispensable to the young practitioner, and, forsooth, they would not be out of place among the every-day books of some of the older workers. They make no pretense to being exhaustive works on general surgery. They are just what their titles indicate them to be—excellent works on special subjects. The great fault of general surgery works is that an effort is made to cover too much ground. Such volumes as these cover their subjects thoroughly and intelligently without an accompanying brass band display of pathological findings and surmises. In his work on Railway Surgery, Dr. Herrick starts off well and properly. He knows his subject, he tells what he knows and he tells it in an easy manner. There are none of the throes of literary genius visible. He briefly sketches the history and statistics of railroad operations and attending casualties. He advises regarding first aid and explains hospital car management. The manner in which various railroad wounds are received, is shown by photo-engravings, and the forces at play in wound production are shown by engravings and explained in the text. From this point on, matters are handled in a masterly manner. The nature, appearance, history and diagnosis of railway wounds includes many slow and fast train injuries and in this section, which is handsomely illustrated by half-tone reproductions, are many interesting statements of the result of injuries. Following this the author treats of shock, primary treatment of wounds, transportation of the wounded, the surgeon's outfit, anesthetics. Then are taken up definite injuries: cuts, bruises, burns, scalds, sprains, dislocations, with diagnosis, treatment and prognosis; wounds of the face and scalp; fractures of the skull, ribs, vertebræ, pelvis, jaw and clavicle and of the extremities, with method of treatment and prognosis, showing casts and splint arrangements in half-tone plates. One of the most important chapters is that on injuries to the hands and fingers and why, when, where and how to amputate. The illustrations here are most instructive. They teach in small space a great deal more than could be taught in pages and pages of writing.

Internal injuries and spinal cord injuries are carefully considered and then Dr. Herrick goes into the question of traumatic neurasthenia —a most important one for the consideration of a railroad surgeon. The closing chapters deal with jurisprudence in railway surgery, the examination of employees and car sanitation and disinfection.

---

Dr. Pilcher's previous work on wound treatment is so well-known that this new up-to-date volume needs nothing at the hands of a reviewer in the way of introduction and mighty little in the way of commendatory writing. The work is summed up in a sentence in the introduction: "The stage of novelty and of disputatious discussions as to the fundamental facts of the new surgery has passed. The chief interest of to-day gathers about questions of how to most perfectly bring practicable results in the ever varying and widely differing conditions in which wounds present themselves for treatment."

The work is divided into parts dealing with wounds in general and special wounds. Under the first heading the principles and practice of wound treatment are dealt with, and there is everything here about wounds, their treatment, complications and repair. Nothing is overlooked, everything is handled in a most masterly manner, and one feels wiser after reading. The special wounds of the second part of the book include gunshot wounds and wounds of muscles, tendons, nerves, blood-vessels and bones. The chapters on wounds of special regions are interesting from every view point.

Method, apparatus and positions are excellently illustrated throughout the work, especial attention being given to gun-shot wounds and the balls that produce them.

There is just enough pathology and bacteriology given to round out the general scope of the work and give the volume a completeness which otherwise would be lacking. Whatever is known of wounds and wound treatment can be found in Dr. Pilcher's work, and as a working reference book it will prove simply invaluable.

